# Diagnosis and management of non-dialysis chronic kidney disease in ambulatory care: a systematic review of clinical practice guidelines

**DOI:** 10.1186/s12882-018-1048-5

**Published:** 2018-10-11

**Authors:** Gesine F C Weckmann, Sylvia Stracke, Annekathrin Haase, Jacob Spallek, Fabian Ludwig, Aniela Angelow, Jetske M Emmelkamp, Maria Mahner, Jean-François Chenot

**Affiliations:** 1 Department of General Practice, Institute for Community Medicine, University Medicine Greifswald, Fleischmannstr. 6, 17475 Greifswald, Germany; 2 Department of Internal Medicine A, Nephrology Dialysis and Hypertension, University Medicine Greifswald, Greifswald, Germany; 3 Department of Public Health, Brandenburg University of Technology Cottbus-Senftenberg, Senftenberg, Germany; 4 Department II – Cardiology, Clinic for Internal Medicine, Pulmonology and General Internal Medicine, DRK-Krankenhaus Teterow, Teterow, Germany; 5 Faculty of Applied Health Sciences, European University of Applied Sciences, Rostock, Germany

**Keywords:** Chronic kidney disease, Management, Clinical practice guideline, Systematic review

## Abstract

**Background:**

Chronic kidney disease (CKD) is age-dependent and has a high prevalence in the general population. Most patients are managed in ambulatory care. This systematic review provides an updated overview of quality and content of international clinical practice guidelines for diagnosis and management of non-dialysis CKD relevant to patients in ambulatory care.

**Methods:**

We identified guidelines published from 2012-to March 2018 in guideline portals, databases and by manual search. Methodological quality was assessed with the Appraisal of Guidelines for Research and Evaluation II instrument. Recommendations were extracted and evaluated.

**Results:**

Eight hundred fifty-two publications were identified, 9 of which were eligible guidelines. Methodological quality ranged from 34 to 77%, with domains “scope and purpose” and “clarity of presentation” attaining highest and “applicability” lowest scores. Guidelines were similar in recommendations on CKD definition, screening of patients with diabetes and hypertension, blood pressure targets and referral of patients with progressive or stage G4 CKD. Definition of high risk groups and recommended tests in newly diagnosed CKD varied.

**Conclusions:**

Guidelines quality ranged from moderate to high. Guidelines generally agreed on management of patients with high risk or advanced CKD, but varied in regarding the range of recommended measurements, the need for referrals to nephrology, monitoring intervals and comprehensiveness. More research is needed on efficient management of patients with low risk of CKD progression to end stage renal disease.

**Electronic supplementary material:**

The online version of this article (10.1186/s12882-018-1048-5) contains supplementary material, which is available to authorized users.

## Background

Chronic kidney disease (CKD) has a high prevalence in the general population and is defined as kidney damage or glomerular filtration rate (GFR) < 60 mL/min/1.73 m^2^ for 3 months or more, irrespective of cause [1, 2]. In the general adult population, CKD stages 3–5 have a prevalence of up to 10%. Because kidney function declines with age, the prevalence of CKD is higher in the elderly population, with ca. 40–50% in the age group of over 85 years old meeting the criteria for CKD [3–6].

Most important risk factors for CKD are diabetes and hypertension [7]. CKD is associated with an increased risk of cardiovascular disease and can progress to end-stage renal disease [8]. However, only a small minority of patients with CKD will progress to end stage renal disease (ESRD) during their lifetime [9]. Medical care of non-dialysis patients is mostly provided by primary care providers.

Observational studies on management of chronic kidney disease in primary and ambulatory care, have concluded that management of patients with CKD could benefit from the implementation of clinical practice guidelines [3, 10–18]. Fundamental to the development of clinical practice guidelines is the review of existing evidence based guidelines.

The aim of this review is to compare quality, scope, consistency and methodological rigor of clinical practice guidelines on diagnosis and management of non-dialysis CKD.

## Methods

This is a systematic review of clinical practice guidelines on diagnosis and management of CKD in adult patients in ambulatory care.

This systematic review was prospectively registered as CRD42016016939 in the PROSPERO registry.

### Search strategy

A systematic search was performed to identify all relevant contemporary guidelines. The search strategy was confined to guidelines on diagnosis and management of adult non-pregnant ambulatory patients with chronic, non-dialysis CKD (GFR ≥30 ml/min/1.73m^2^) that had been issued or updated between January 1, 2012 and March 20 2018. The search was limited to clinical practice guidelines in the languages English, French, Dutch/Flemish and German. Only guidelines issued in industrialized countries were considered eligible to ensure comparability.

#### Guideline portals

We performed a search using the following guideline portals:

- Guidelines-International-Network (G-I-N) [www.g-i-n.net].
- NHS Centre for Reviews and Dissemination (CRD) [19]
- National guideline Clearinghouse [10]
- Haute Autorité de Santé (HAS) [20]
- Ärztliches Zentrum für Qualität in der Medizin (AEZQ) [21]
- Arbeitsgemeinschaft der Wissenschaftlichen Medizinischen Fachgesellschaften (common working group of scientific medical Specialty Associations, AWMF) [www.awmf.org]

These guideline portals were searched with the terms:

**“chronic kidney disease”**

for the English language portals and

**“chronische Niereninsuffizienz”**

for the German language portals

#### Database

A search of the database Pubmed was performed with the algorithm (last update March 20 2018):

**(((((((((“2012/01/01”[Date - Completion]**: **“3000”[Date - Completion])) AND ((((((clinical practice guideline) OR clinical practice guidelines) OR guideline) OR guidelines[MeSH Terms])) AND (((chronic kidney disease) OR CKD) OR chronic kidney insufficiency[MeSH Terms])))) NOT (child OR children or adolescents or infants)) NOT (dialysis OR intensive care))))) NOT (tumor OR malignancy)**

Sciencedirect was searched with **“guideline” AND “chronic kidney disease”** for the years 2012–2018, article type: “practice guidelines”.

#### Google search

A targeted search for eligible clinical practice guidelines was performed for the following European countries: Belgium, Denmark, Finland, France, Iceland, Ireland, the Netherlands, Norway, Sweden Switzerland and the United Kingdom. From the non-European countries a search was performed for Australia, Canada, Israel, New Zealand, South Africa and the United States of America. We used the following mesh terms in English and in the language of the country in question:

**“<country>” AND “kidney” AND “guideline”.**

to search the World Wide Web with the Google browser and scanned the first 5 pages for eligible guidelines. If no guidelines were found, the nephrological society in this country was identified and its website was searched for information concerning national guidelines. If no such information was listed on the website, a request for information was sent to the organization.

#### Manual search

We conducted a manual search for additional guidelines in the reference lists of identified guidelines.

### Selection of guidelines

For the selection of eligible guidelines we used predefined in- and exclusion criteria.

Inclusion criteria (Table 1).

**Table 1:** Inclusion and exclusion criteria for clinical guidelines on chronic kidney disease

Inclusion criteria	Excluson criteria:
guideline issued in an industrialized country	relevance limited to subspecialty or subtheme
guideline is relevant to management of patients with CKD	relevance is limited to acute renal insufficiency
guideline is targeted to adult patients	target group of children
guideline is available in one of the following languages: Dutch/Flemish, English, French, German	relevance is limited to pregnancy or childbirth
guideline is relevant to ambulatory patients	relevance is limited to KDIGO stage 4 and above
	relevance is limited to patients on dialysis
	relevance is limited to kidney transplant patients
	relevance is limited to inpatients

*CKD* Chronic Kidney Disease

A prior systematic guideline review had identified and evaluated guidelines on early CKD up to 2011 [8]. For this reason and to ascertain compliance of the guidelines with current state of research, we limited the search to guidelines that had been issued or updated since 2012. When guideline updates had been issued, we included the most recent update. Supplementary information was considered when the guideline referred to this information.

### Quality assessment

All eligible guidelines were assessed by 2 authors independently, using the AGREE-II instrument for guideline quality assessment [22]. The AGREE instrument measures methodological rigor in guideline development [22]. The AGREE-II instrument consists of 6 domains, consisting of 23 items and one overall assessment [22]. The content of the different domains of this instrument are listed in Additional file 1. Guidelines were rated by 2 independent researchers (AA, JFC, JME, FL, SS, GW). Scores indicate the extent to which a predefined quality dimension has been fulfilled and vary on an ordinal scale from 1 “strongly disagree” to 7 “strongly agree”.

Individual AGREE-II-items were discussed in a consensus meeting between the first 2 reviewers, when a difference of 3 or more points was detected in individual item ratings, to allow for correction of false allocation of the ratings. A third reviewer would be appointed when 3 of the domains had an average item score standard deviation of ≥1,5 or if one of the domains had a standard deviation of > 2 [22].

Scaled domain scores were automatically calculated by an integrated program in the online version of the AGREE-II instrument: (Obtained score – Minimum possible score) / (Maximum possible score – Minimum possible score) [22]. Overall guideline scores were calculated as weighted mean of the domain scores.

### Data extraction

A synthesis of recommendations of the selected guidelines regarding content, consistency and strengths of recommendations, as well as level of evidence, was compiled by extracting recommendations, strength or recommendation and level of evidence in a predefined form. Recommendations were inserted into the form by AH, CK, FL and GW and grouped by domain, to enable the identification of discrepancies and similarities. Domains were: prevention and screening, diagnostic tests in newly diagnosed CKD, monitoring, referral criteria, blood pressure and anemia management, and a group of miscellaneous recommendations.

## Results

### Selection of guidelines

**Fig. 1:**
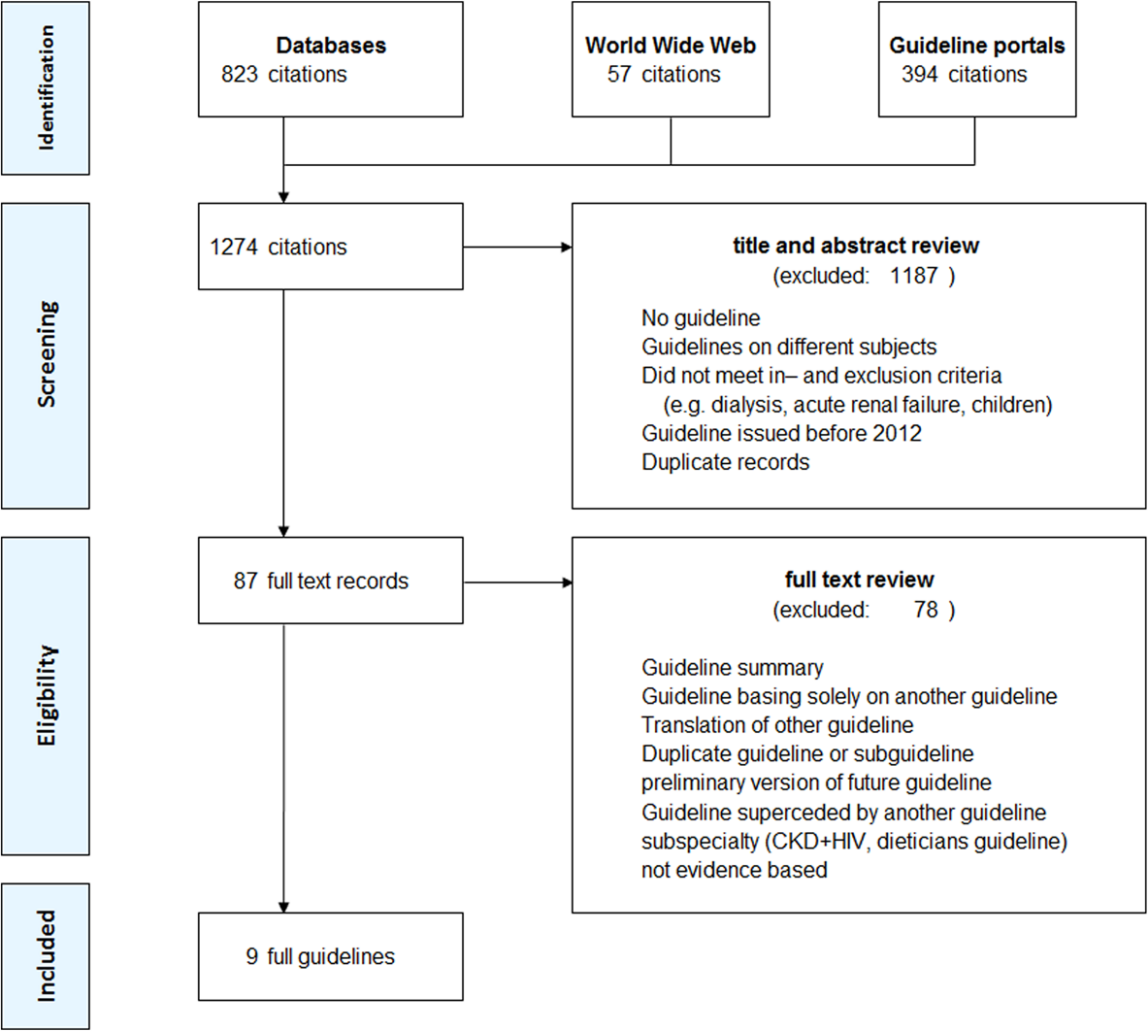
Flow diagram of results of literature search and guideline selection

We identified 1274 potentially relevant records. We excluded 1187 after title and/or abstract review. Eighty-seven potentially relevant guidelines were included in full text review (Fig. 1). Of these, 76 guidelines did not meet eligibility criteria, one was a duplicate and 1 a preliminary version of an unpublished guideline. After full text review, we retained 9 guidelines and one USPSTF statement (Table 2) [23, 24].

**Table 2:** Characteristics of included guidelines and one statement

	country	Issueing organization	name of guideline	initial release	revisions	target patients	target users/setting	evidence base	grading of evidence	
									LoE	GoR
CEBAM	Belgium	Belgian Centre for Evidence Based Medici, Cochrane Belgium	Chronische Niereninsufficiëntie	2012		adult patients (over 18 years of age) with chronically diminished kidney function	general practitioners	systematic guideline review, additional systematic searches	GRADE	
ACP	USA	American College of Physicians	Screening, Monitoring, and Treatment of Stage 1 to 3 Chronic Kidney Disease: A Clinical Practice Guideline From the American College of Physicians	2013		target patient population for screening is adults, and the target population for treatment it is adults with stage 1 to 3 CKD	clinicians	systematic review	American College of Physicians grading system, adapted from GRADE	
HAS	France	Haute Autorité de Santé	Guide de parcours de soins Maladie Rénale Chronique de l’adulte	2012		Adult patients with chronic kidney disease. Excluded: patients with end stage renal disease, dialysis or transplantation, inpatients.	General practitioners, dieticians, nurses, pharmacists, etc., and may also concern other health professionals (Nephrologists, cardiologists, diabetologists, physiotherapists, psychologists)	unclear, existing recommendations, expert opinion	no formal grading of evidence or level of recommendation	
KDIGO	USA	Kidney Disease Improving Global Outcomes	KDIGO 2012 Clinical Practice Guideline for the Evaluation and Management of Chronic Kidney Disease	2012		individuals at risk for or with CKD	Providers: Nephrologists (adult and pediatric), dialysis providers(including nurses), Internists, and pediatricians.patients: Adult and pediatric individuals at risk for or with CKD. Policy Makers: Those in related health fields.	systematic review	GRADE	
KHA-CARI	Australia, New Zealand	Kidney Health Australia, Caring for Australasians with Renal Impairment	Early Chronic Kidney Disease	2013		patients with kidney disease in Australia & New Zealand, patients with early chronic kidney disease	clinicians and health care workers	systematic review	GRADE	
BCMA	Canada	British Columbia Medical Association	Chronic Kidney Disease - Identification, Evaluation and Management of Adult Patients	2014		adults aged ≥19 years at risk of or with known chronic kidney disease	The primary audience for BC Guidelines is British Columbia physicians, nurse practitioners, and medical students. However, other audiences such as health educators, health authorities, allied health organizations, pharmacists, and nurses may also find them to be a useful resource	not described	no formal grading of evidence or level of recommendation	
UMHS	USA	University of Michigan Health System	Management of Chronic Kidney Disease	2005	Interim/minor revision: March, 2014 June, 2016	adults with chronic kidney disease	clinicians, primary care poviders	systematic review	GRADE, not formally stated	
VA-DoD	USA	Department of Veterans Affairs, Department of Defense	VA/DoD Clinical Practice Guideline for the Management of Chronic Kidney Disease in Primary Care	2014	–	adults 18 years or older with CKD 1–4 without kidney transplant	primary care providers	systematic review	GRADE	
NICE	UK	National Institute of Health and Care Excellence	Early identification and management of chronic kidney disease in adults in primary and secondary care	2014	Update 2015	Adults 18+ with or at risk of developing chronic kidney disease	Healthcare professionals Commissioners and providers People with chronic kidney disease and their families and carers	systematic review	NICE	
USPSTF	USA	United States Preventive Services Task Force	Final Recommendation statement, Chronic Kidney Disease: Screening	2012		asymptomatic adults without diagnosed CKD	clinicians	probably systematic review “The USPSTF reviewed evidence on screening for CKD, including evidence on screening, accuracy of screening, early treatment, and harms of screening and early treatment.”	one recommendation, not graded	

*GoR* grade of recommendation, *LoE* level of evidence

### Quality assessment

The quality of the guidelines was assessed with the Appraisal of Guidelines for Research and Evaluation instrument (AGREE-II) [22]. Interrater variability was low for all guidelines. Domains with high average scores were “scope and purpose” with 58–100% and “clarity of presentation” 53–100%. Lowest average score was found for “applicability” with 4–60% average score whereas editorial independence had a highly variable score with 0–96%. Guidelines achieving ratings of > 70% over all domains were the NICE guideline and the KDIGO guideline, with weighted mean domain scores of 75% and 73% respectively. KHA-CARI, BCMA and HAS guidelines received the lowest scores (Table 3). No correlation was found between year of publication and domain score, but total score correlated with rigor of development (data not shown).

**Table 3:** Results of guideline assessment with AGREE

	CEBAM	HAS	ACP	KDIGO	KHA-CARI	BCMA	NICE	UMHS	VA-DoD	mean	range	
Scope and Purpose	72%	75%	81%	100%	61%	58%	75%	67%	89%	75%	58%	100%
Stakeholder Involvement	53%	75%	8%	89%	25%	31%	67%	39%	61%	50%	8%	89%
Rigour of Development	55%	19%	53%	70%	29%	17%	77%	40%	59%	47%	17%	77%
Clarity of Presentation	72%	53%	69%	100%	61%	78%	81%	69%	67%	72%	53%	100%
Applicability	50%	15%	4%	29%	13%	27%	60%	25%	10%	26%	4%	60%
Editorial Independence	96%	0%	88%	79%	67%	25%	88%	71%	29%	60%	0%	96%
weighted mean	61%	38%	42%	73%	34%	36%	75%	45%	54%	51%	34%	75%

Selected general clinical practice guidelines were rated with the AGREE-II instrument [22]. Scaled domain scores were calculated as percentage of the difference between the minimum possible score and the maximum possible score for a particular domain. Belgisch Centrum voor Evidence Based Medicine (CEBAM), Haute Autorité de Santé (HAS), American College of Physicians (ACP), Kidney Disease Improving Global Outcomes (KDIGO), Caring for Australians with Renal Insufficiency (KHA-CARI), British Colombia Medical Association (BCMA), National Institute of Health and Care Excellence (NICE), University of Michigan Health System (UMHS), Department of Veteran’s Affairs (VA-DoD)

#### Scope and purpose

Missing items included incomplete description of health questions and imprecise objectives. KDIGO was the only guideline scoring 100% for this domain, whereas VA-DoD and ACP scored 89% and 81% respectively.

#### Stakeholder and patient involvement

Several guidelines incompletely described the target user group. Guideline development groups were not always defined and often did not include methodologists, primary care physicians and health care workers other than physicians.

#### Rigor of development

Systematic evidence search and selection were incompletely described in several guidelines. Strengths and limitations of the evidence were not rigorously discussed by several guidelines. Health benefits and side effects were inconsistently considered in formulating recommendations. Only NICE described a structured strategy for formulating recommendations. External reviews were incompletely reported by most guidelines. Several guidelines incompletely described an updating procedure.

#### Clarity of presentation

Wording of recommendations was mostly unambiguous, but treatment alternatives where inconsistently addressed. The option abstaining form therapy was only mentioned by NICE.

#### Applicability

Facilitators and barriers and implementation strategies were incompletely addressed in most guidelines. No guideline described formal tools for barrier analysis. Only NICE consistently considered resource implications of recommendations and auditing and monitoring criteria. KDIGO provided no recommendations for implementation since it is intended to be a template for national adaptations.

#### Editorial Independence

Independence of the funding body was incompletely reported in several guidelines and two guidelines did not report conflicts of interest (Additional file 1).

### Recommendations

#### Definition

The definition of CKD in the included guidelines was congruent with the KDIGO definition of CKD as abnormalities of kidney structure or function with albuminuria or GFR < 60 ml/min/1.73m^2^ for > 3 months [25].

CEBAM and USPSTF restricted the definition to decreased kidney function persisting for more than 3 months. None of the guidelines provided a description of relevant structural kidney abnormalities.

#### Prevention

General lifestyle recommendations like weight management and sodium restriction for CKD prevention were mentioned only by KHA-CARI with medium grades of recommendation and low levels of evidence (Table 4) [26]. Other guidelines’ lifestyle recommendations were aimed solely at persons with established CKD [26].

**Table 4:** Recommendation summary – Prevention and screening

	CEBAM	USPTF	ACP	HAS	KHA-CARI	BCMA	UMHS	VA-DoD	NICE
	2012	2012	2013	2013	2013	2014	2014	2014	2015
Prevention and Screening									
Prevention									
weight management					▪				
sodium restriction					▪				
protein restriction					–				
smoking abstinence					▪				
reducing excessive alcohol intake					▪				
physical exercise					▪				
Screening									
asymptomatic		–	–						–
diabetes	▪			▪	▪	▪	▪	▪	▪
hypertension				▪	▪	▪		▪	▪
cardiovascular disease	▪			▪	▪	▪	▪	▪	▪
acute kidney injury				▪				+	▪
structural renal tract disease, renal calculi, prostate hypertrophia				▪					▪
systemic illness (e.g. SLE, HIV)				▪					▪
positive family history					▪	▪		▪	▪
hematuria	▪								▪
nephrotoxic drugs				▪					▪*
smoking					▪				
age							> 55		–
gender									–
ethnicity					▪	▪		▪	–
obesity				▪	▪				–
occupational hazards				▪				▪	
socioeconomic disadvantage					▪				

▪ recommendation, − negative recommendation, * including NSAID

American College of Physicians (ACP), Belgisch Centrum voor Evidence Based Medicine (CEBAM), British Columbia Medical Association (BCMA), Department of Veteran’s Affairs (VA-DoD), Haute Autorité de Santé (HAS), Kidney Disease Improving Global Outcomes (KDIGO), Kidney Health Australia - Caring for Australasians with Renal Impairment (KHA-CARI), National Institute of Health and Care Excellence (NICE), University of Michigan Health System (UMHS)

#### Screening

None of the guidelines recommended screening for CKD in asymptomatic persons without risk factors and NICE, ACP and USPTF guidelines explicitly advised against it (Table 4). Most guidelines recommended screening in persons with risk factors like diabetes, cardiovascular risk, or positive family history for ESRD. Notably, the UMHS guideline considered age a risk factor and recommended screening persons over 55 [23].

#### Diagnostic tests in newly diagnosed CKD

Serum creatinine, eGFR and proteinuria testing were recommended most often (Table 5). HAS and KHA-CARI issued detailed recommendations for more extensive testing. HAS stated that some of the tests should only be ordered if recommend by a nephrologist.

**Table 5:** Recommendation summary - diagnostic tests in newly diagnosed CKD

	CEBAM	ACP	HAS	KDIGO	KHA-CARI	BCMA	UMHS	VA-DoD	NICE
	2012	2013	2013	2013	2013	2014	2014	2014	2015
Diagnostic Tests in newly diagnosed CKD									
clinical blood tests									
blood pressure					▪				
serum creatinine			▪	▪	▪				
(e)GFR (creatinine)	*		▪		▪	▪	▪	▪	▪
blood count			▪		▪				
serum urea			i		▪				
serum uric acid			▪						
serum albumin			i		▪				
serum electrolytes			▪		▪				
serum glucose			▪		▪				
lipids			▪		▪				
serum cystatin C				i					
eGFR (cystatin C)									i
clearance				i					
HbA1c									
serum calcium			▪		i				
serum phosphate					i				
serum phosphorus			i						
serum PTH			▪		i				
serum 25-hydroxy-Vitamin D			▪		i				
iron					i				
serum electrophoresis			i		i				
ANA			i		i				
anti-ENA					i				
complement			i		i				
Hepatitis-B serology					i				
Hepatitis-C serology					i				
HIV-serology					i				
anti-GBM			i		i				
ANCA			i		i				
inulin									i
^51^Cr-EDTA									i
^125^I-iothalamate									i
iohexol									i
urine tests									
albuminuria			▪	▪	i		▪	▪	–
proteinuria - reagent strips									- ***
urine albumin-creatinin-ratio (ACR)	▪**				i	▪			n
urine protein-creatinin ratio (PCR)	▪**								i
urine leucocytes			▪						
hematuria			▪	(▪) ****					unclear*****
urine microscopy					▪				(−)
24 h urine			i						
urine electophoresis					i				
imaging									
renal ultrasound	i		▪		▪		i	▪	i
bladder ultrasound			i						
MRI									
CT									
Angiography									
renal artery doppler			i				i		
invasive									
kidney biopsy			i						

▪ recommendation, − negative recommendation, i: when indicated, *implicitly mentioned, **ACR or PCR, ***unless able to detect microalbuminuria, ****no explicitly formulated recommendation, but mentioned in background and a flow diagram, *****opportunistic detection

*ANA* anti-nuclear antibodies, *anti-ENA* anti extractable nuclear antibodies, *ANCA* anti-neutrophil cytoplasmic antibodies, *anti-GBM* anti-glomerular basement membrane antibodies, *eGFR* estimated glomerular filtration rate, *PTH* parathyroid hormone

American College of Physicians (ACP), Belgisch Centrum voor Evidence Based Medicine (CEBAM), British Columbia Medical Association (BCMA), Department of Veteran’s Affairs (VA-DoD), Haute Autorité de Santé (HAS), Kidney Disease Improving Global Outcomes (KDIGO), Kidney Health Australia - Caring for Australasians with Renal Impairment (KHA-CARI), National Institute of Health and Care Excellence (NICE), University of Michigan Health System (UMHS)

#### Monitoring

Several guidelines issued recommendations on monitoring. Monitoring intervals were mostly congruent with KDIGO recommendations, but NICE recommended less frequent monitoring in early CKD (Table 6). Monitoring recommendations included eGFR and proteinuria, but several guidelines recommended monitoring other parameters such as weight, cardiovascular risk (BCMA, HAS), smoking status and psychosocial health (BCMA). Only HAS and BCMA and ACP explicitly recommended monitoring blood pressure and only BCMA and ACP recommended reviewing medication. BCMA recommended more extensive blood testing.

**Table 6:** Recommendation summary – Monitoring recommendations for patients with established CKD

	CEBAM	ACP	HAS	KDIGO	KHA-CARI	BCMA	UMHS	VA-DoD	NICE
	2012	2013	2013	2013	2013	2014	2014	2014	2015
Monitoring patients with known CKD									
frequency (times /year)									
G1/A1	1			1		1	1		≤1
G1/A2	1			1		1	1		1
G1/A3	1			2		2	2		≥1
G2/A1	1			1		1	1		≤1
G2/A2	1			1		1	1		1
G2/A3	2			2		2	2		≥1
G3a/A1	2			1		1	1		1
G3a/A2	2			2		2	2		1
G3a/A3	2			3		3	3		2
G3b/A1	2			2		2	2		≤2
G3b/A2	2			3		3	3		2
G3b/A3	≥4			3		3	3		≥2
G4/A1	≥4			3		3	4**		2
G4/A2	≥4			3		3	3		2
G4/A3	≥4			≥4		≥4	≥4		3
G5/A1	≥4			≥4		≥4	≥4		4
G5/A2	≥4			≥4		≥4	≥4		≥4
G5/A3	≥4			≥4		≥4	≥4		≥4
parameter									
blood pressure	*	▪	▪	*		▪	*		*
weight						▪			
(e)GFR	▪	▪	▪	▪		▪	▪		▪
albuminuria/proteinuria/ACR	▪	▪	▪	▪		▪	▪		▪
complete blood count						▪			
iron saturation						▪			
HbA1c						▪			
serum calcium						▪			
serum phosphorus						▪			
serum potassium						i	i		
serum albumin						▪			
complications	▪								
inulin									i
51Cr-EDTA									i
125I-iothalamate									i
iohexol									i
cardiovascular risk			▪			▪➢			
smoking status						▪			
medication		▪				▪			
psychosocial health						▪			

▪ recommendation, − negative recommendation, i: when indicated, *not specifically mentioned, but obvious from the context (e.g. blood pressure targets), **probably transcription error, ➢ refers to British Columbian guideline “Cardiovascular disease - primary prevention”

Stages of CKD: G1, glomerular filtration rate of ≥90 ml/min/1.73m^2^; G2, 60–89 ml/min/1.73m^2^; G3a, 45–59 ml/min/1.73m^2^; G3b, 30–44 ml/min/1.73m^2^; G4, 15–29 ml/min/1.73m^2^; G5, < 15 ml/min/1.73m^2^

Albuminuria stages of CKD: A1, albumine-creatinine-ratio < 3 mg/mmol; A2, 3–30 mg/mmol; A3, > 30 mg/mmol

*ACR* albumin-creatinine-ratio, *eGFR* estimated glomerular filtration rate, *HbA1c* glycated hemoglobin, *51Cr-EDTA* chromium-51-ethylenediaminetetraacetic acid

American College of Physicians (ACP), Belgisch Centrum voor Evidence Based Medicine (CEBAM), British Columbia Medical Association (BCMA), Department of Veteran’s Affairs (VA-DoD), Haute Autorité de Santé (HAS), Kidney Disease Improving Global Outcomes (KDIGO), Kidney Health Australia - Caring for Australasiansians with Renal Impairment (KHA-CARI), National Institute of Health and Care Excellence (NICE), University of Michigan Health System (UMHS)

#### Referral criteria

Most guidelines recommend referring patients to a nephrologist if GFR falls below 30 ml/min/1.73m^2^ (Table 7). HAS recommends a higher cut-off value of 45 ml/min/1.73m^2^. Guidelines generally agreed in recommending referral in case of proteinuria. Only few guidelines differentiated between low-threshold consultation (NICE, KHA-CARI) or co-management versus long-term referral for management of (advanced) CKD. Multidisciplinary or co-management was mentioned by several guidelines. Only CEBAM explicitly described the role of general practitioners (GP) and recommended GP to be responsible for detecting and monitoring CKD, detecting complications and treating cardiovascular risk.

**Table 7:** Recommendation summary - referral criteria

		CEBAM	ACP	HAS	KDIGO	KHA-CARI	BCMA	UMHS	VA-DoD	NICE
		2012	2013	2013	2013	2013	2014	2014	2014	2015
Referral Criteria										
general	consider individual preferences					▪				▪
	consider individual comorbidities			▪						▪
	cooperation or multidisciplinary care	▪			i	▪			▪	▪
	routine follow-up after referral by patient’s GP					▪				▪
nephrologist	GFR < 60 ml/min/1,73m^2^									
	GFR < 45 ml/min/1,73m^2^	i		▪						
	GFR < 30 ml/min/1,73m^2^	▪			▪	▪	▪	▪	▪	▪
	ACR > 30 mg/mmol	▪*			▪		▪			+ hematuria
	ACR ≥70 mg/mmol			▪						i#
	proteinuria > 3500 mg/day								▪	
	hematuria				i	▪*				
	urinary cell casts						▪			
	constitutional symptoms						▪			
	CKD progression	▪		▪	▪	▪	▪		▪	▪
	poorly controlled hypertension				▪	▪	▪			▪
	electrolyte disturbance			i	▪		▪		▪	
	anemia			i				▪	▪	
	metabolic complications			i					▪	
	complications			i				i		
	nephrolythiasis				▪				▪	
	suspected renal artery stenosis	▪								▪
	genetic etiology of CKD				▪		▪			▪
	rare etiology of CKD									▪
	etiology requiring specialist care								▪	
	unclear etiology						i	i	▪	
	1-year ESRD-risk of ≥10%				▪					
	indication for dialysis or transplant				▪		▪			▪
urologist	renal outflow obstruction	▪								▪
diabetologist	diabetic nephropathy						▪			▪
dietician	eGFR< 60 ml/min/1,73m^2^			▪	i					i
inpatient treatment	complications									▪
	hypertensive crisis									▪
	unknown etiology									▪

▪ recommendation, i: when indicated *in combination with KDIGO stage A3, # unless caused by diabetes and properly treated

*ACR* albumin-creatinine-ratio, *CKD* chronic kidney disease, *ERSD* end stage renal disease, *GFR* glomerular filtration rate, *GP* general practitioner, *HbA1c* glycated hemoglobin

American College of Physicians (ACP), Belgisch Centrum voor Evidence Based Medicine (CEBAM), British Columbia Medical Association (BCMA), Department of Veteran’s Affairs (VA-DoD), Haute Autorité de Santé (HAS), Kidney Disease Improving Global Outcomes (KDIGO), Kidney Health Australia - Caring for Australasiansians with Renal Impairment (KHA-CARI), National Institute of Health and Care Excellence (NICE), University of Michigan Health System (UMHS)

#### Blood pressure

All guidelines recommended blood pressure targets of < 140/90 mmHg, with lower targets of 130/80 mmHg for patients with diabetes or albuminuria. As first line treatment, guidelines consistently recommended renin-angiotensin system antagonists, whereas diuretics, betablockers and calcium antagonists were mentioned as second line options by KHA-CARI and BCMA. Combining angiotensin converting enzyme inhibitors with angiotensin receptor blockers was explicitly not recommended by several guidelines (Table 8).

**Table 8:** Recommendation summary - blood pressure management

		CEBAM	ACP	HAS	KDIGO	KHA-CARI	BCMA	UMHS	VA-DoD	NICE
		2012	2013	2013	2013	2013	2014	2014	2014	2015
Blood pressure management										
BP monitoring intervals						▪				
individualized BP targets					▪		▪			▪
BP target	< 140/90	▪		▪	▪	▪	▪	▪	▪	▪
BP target in diabetics	< 140/90							GP		
	< 140/80									
	< 130/80					▪				▪
BP target in ≥ microalbuminuria	< 140/90							▪		
	< 130/80				▪	▪		i		▪
medication	renin-angiotensin system antagonist						i ➢			i
	ACEI	i	i		i	i	i	▪	i	
	ARB		i		i	i	i	▪	i	
	combination of ACEI + ARB				–	–			–	–
	combination of ACEI/ARB + direct renin inhibitor					–		–	–	
	diuretics					i	i			
	β-blocker					i	i			
	calcium channel blocker					i	i			
	side effects	▪								

▪ recommendation, − negative recommendation, i: when indicated, ➢ recommendations in KDIGO BP guideline, *ACEI* angiotensin converting enzyme inhibitor, *ARB* angiotensin receptor blocker, *BP* blood pressure, *DM* diabetes mellitus, *ev* insufficient evidence for recommendation, GP: identical blood pressure targets as general population, n.a.: not applicable

American College of Physicians (ACP), Belgisch Centrum voor Evidence Based Medicine (CEBAM), British Columbia Medical Association (BCMA), Department of Veteran’s Affairs (VA-DoD), Haute Autorité de Santé (HAS), Kidney Disease Improving Global Outcomes (KDIGO), Kidney Health Australia - Caring for Australasiansians with Renal Impairment (KHA-CARI), National Institute of Health and Care Excellence (NICE), University of Michigan Health System (UMHS)

#### Anemia

Several guidelines issued recommendations on diagnosis, monitoring or treatment of anemia. Therapeutic targets for serum hemoglobin (6.8 moll/l; Hb, 11 g/dl) were lower than the normal values (7,5–8.1 moll/l;12-13 g/dl) (Table 9). Except for HAS and to a lesser extent CEBAM, guidelines did not contain details on the treatment of renal anemia and instead referred to specific guidelines on this topic [27–29]. Only HAS explicitly recommended avoiding blood transfusion in patients who may need kidney transplant.

**Table 9:** Recommendation summary - anemia management

		CEBAM	ACP	HAS	KDIGO	KHA-CARI	BCMA	UMHS	VA-DoD	NICE
		2012	2013	2013	2013	2013	2014	2014	2014	2015
Management of anemia										
diagnosis	definition	▪			▪			▪		▪
	lower limit in g/dl	11			M: 13, F: 12			M: 13, F: 12		11
monitoring	monitor for anemia	▪		▪	▪		i	▪		
	tests	▪			▪			▪		
	frequency (per year)	individual			1–4					
initial evaluation								▪		
treatment options	iron	▪		i					▪	
	erythropoetin	▪		i						
	nutritional supplements			i						
	androgens									
	blood transfusion			−/i*						
treatment	indications			▪						
	target values			▪						
	monitoring			▪						
	erythropoietine resistance			▪						
	referral			▪						

▪ recommendation, − negative recommendation, F: female, M: male, i: when indicated, *Transfusions should be avoided (risk of allo-immunization). The only indications are symptomatic anemia in patients with an associated risk factor; acute worsening of anemia by blood loss (hemorrhage, surgery), hemolysis or resistance to erythropoietin. A search for anti-HLA antibodies should be performed before and after any transfusion in patients waiting for kidney transplant

American College of Physicians (ACP), Belgisch Centrum voor Evidence Based Medicine (CEBAM), British Columbia Medical Association (BCMA), Department of Veteran’s Affairs (VA-DoD), Haute Autorité de Santé (HAS), Kidney Disease Improving Global Outcomes (KDIGO), Kidney Health Australia - Caring for Australasiansians with Renal Impairment (KHA-CARI), National Institute of Health and Care Excellence (NICE), University of Michigan Health System (UMHS)

#### Other subjects

Some guidelines issued recommendations on CKD-mineral bone disorder, patient education, and various issues pertaining to early or advanced CKD (Table 10). ACP and UMHS issued the general recommendation to avoid nephrotoxic medication, whereas NICE recommended using NSAID with caution. Further subjects were treatment objectives for diabetes and congestive heart failure, low protein diet, statin use, hyperuricemia, oral bicarbonate and antiplatelets and anticoagulants.

**Table 10:** Recommendation summary - other subjects

other subjects		CEBAM	ACP	HAS	KDIGO	KHA-CARI	BCMA	UMHS	VA-DoD	NICE
		2012	2013	2013	2013	2013	2014	2014	2014	2015
patient education		▪				▪			▪	▪
diet	protein intake (in g/kg/day)				0.8	0.75–1.0			0.6–0.8	
	no low protein diet < 0.6 g/kg/day					▪				▪
complications	CKD-mineral bone disorder	▪			▪		▪	▪		▪
diabetes	HbA1c target values (in %)				7.0				< 7.0	
	metformin	with caution					avoid/reduce			
	cardiovascular risk					▪				
hyperlipidemia					➢					➢
	statins for cardiovascular risk				i			i		
	statins for CKD progression								–	
	ezetimibe							i		
congestive heart failure		▪			▪					
antigoagulants and antiplatelets		▪			▪	▪				▪
nephrotoxic Medication	geneneral				–			–		
	NSAID									–
vaccinations									▪	
metabolism	hyperuricemia				▪					▪
	oral bicarbonate				▪				▪	▪
nephrotoxic medication			▪					▪		▪

▪ recommendation, − negative recommendation, i: when indicated, ➢ referral to KDIGO and NICE guidelines on lipid management, *CKD* chronic kidney disease, *HbA1c* glycated Hemoglobin, *NSAID* nonsteroidal anti-inflammatory drugs

American College of Physicians (ACP), Belgisch Centrum voor Evidence Based Medicine (CEBAM), British Columbia Medical Association (BCMA), Department of Veteran’s Affairs (VA-DoD), Haute Autorité de Santé (HAS), Kidney Disease Improving Global Outcomes (KDIGO), Kidney Health Australia - Caring for Australasiansians with Renal Impairment (KHA-CARI), National Institute of Health and Care Excellence (NICE), University of Michigan Health System (UMHS)

## Discussion

### Summary of the main results

We identified 9 clinical practice guidelines and one recommendation statement on diagnosis and management of non-dialysis CKD in adults, issued between 2012 and March 2018. Methodological quality of the guidelines ranged between 34 and 77%. All guidelines used the KDIGO definition of CKD. Recommendations for CKD screening were restricted to higher risk groups, but risk factors considered relevant for diagnostic evaluation varied. There was considerable variation of recommended tests in newly diagnosed CKD. Five guidelines published monitoring intervals for established CKD, mostly reflecting the intervals proposed by KDIGO. Monitoring tests were specified by three guidelines. Referral was usually recommended at GFR < 30 ml/min/1.73m^2^ or when indicated by various other risk factors.

### Quality of guidelines

A previous systematic review of clinical practice guidelines, published in 2013, analyzed 15 clinical practice guidelines issued up to 2011 for prevention, detection and management of early CKD [8]. They reported coverage and recommendations, methodological quality varying from 24 to 95%, as measured by the AGREE-II instrument. AGREE-II measures methodological rigor by rating several different aspects of guideline development, but does not appraise the content of recommendations. Low scores imply that important aspects have been omitted. Some guideline developers did not involve primary care physicians, who care for the majority of CKD patients and were target users. Most guidelines did not include the views of health care professionals other than physicians, like nurses or dieticians. Additionally, many guidelines did not describe external review procedures. External review can help to identify potential barriers related to guideline content, organization of health service provision, availability of health services, billing issues and implementation. Few guidelines explicitly discussed barriers and facilitators of guideline implementation. Identifying implementation barriers early can be valuable in resolving potential problems during the guideline development [30].

Most guidelines based recommendations on evidence from systematic literature searches. Limitations of the evidence were not consistently discussed. Only NICE described the formal procedure for formulating recommendations based on the evidence. Providing this information would help to discern recommendations based on clinical trials from those based on consensus [31]. HAS acknowledged the limited evidence and need for consensus on many topics. To reflect scientific development, clinical practice guidelines should be updated periodically, but several guidelines did not provide an expiration date or a procedure for updating.

AGREE assesses whether all treatment options are discussed and trade-offs between benefits and harms are addressed. Only NICE mentioned the option of abstaining from therapy. Potential harms of overdiagnosis and overtreatment should be more consistently incorporated in guidelines [32]. Consideration of individual patient related factors were mentioned in several guidelines. These considerations are especially important for the mostly elderly population affected by CKD. Life expectancy, comorbidities and health priorities are important factors in decisions on testing, therapy and referral for these patients [32]. KDIGO consciously excluded information on resource implications and implementation, considering itself a template for local adaptations. However, although guideline recommendations can have major impact on healthcare cost and health service utilization given the high prevalence of CKD, only few guidelines consistently addressed resource implications. Auditing and monitoring criteria to measure quality of care were only proposed by NICE.

### Content of guidelines

#### Definition and screening

There was no disagreement on the definition of CKD by laboratory tests, but all guidelines fail to precise which structural abnormalities qualify for CKD. NICE and ACP guidelines as well as the USPSTF recommended explicitly against screening of asymptomatic individuals without known risk factors. Screening was recommended for high risk groups in most guidelines, but KHA-CARI used broad definitions for at risk populations like smoking, obesity, socioeconomic disadvantage or age. This can lead to screening situations where health benefits and therapeutic consequences of CKD diagnosis are lacking.

#### Diagnostic tests in newly diagnosed CKD

Main purpose of the initial diagnostic work-up is to establish CKD and rule out emergencies or specifically treatable kidney disorders, e.g. glomerulonephritis. Most guidelines agree on assessing kidney function by eGFR_creatinine_ and proteinuria. Primarily KHA-CARI and HAS, recommend extensive additional diagnostic work-up, mainly to identify possible complications or comorbidities reflecting the epidemiology in specialized nephrology services but not in primary care. As the risk of developing complications like electrolyte disturbances, anemia or CKD-MBD is largely dependent on kidney function, a more differentiated approach according to CKD stage, could lower health service utilization and cost while maintaining quality of care. HAS explicitly stated that testing was aimed to obtain baseline values in some instances. It is debatable whether this set point information has therapeutic consequence.

Assessment of hematuria was inconsistently addressed. While NICE recommended against using urine microscopy, KHA-CARI recommended it. Most primary care providers do not have the skills and equipment to perform urine microscopy. However NICE and KDIGO did not specify when dipstick testing for hematuria is warranted, while most guideline did not address checking for hematuria at all.

#### Monitoring

Guidelines recommending monitoring intervals, generally adopted these from the KDIGO recommendations, although NICE recommended less frequent monitoring for early stage CKD. Monitoring intervals are mainly based on clinical experience and consensus, given a lack of clinical studies evaluating the effect of different monitoring intervals on health outcomes. Guidelines were not always clear which parameters should be monitored continuously. Therefore, individual patients’ preferences, comorbidities and progression risk, should be incorporated in decisions on monitoring frequency*.* Monitoring eGFR and proteinuria was recommended by all guidelines, but the latter might not be necessary if proteinuria has been ruled out.

Other parameters mentioned, were prognostic and etiological factors like diabetes, or laboratory values indicative of complications like CKD-MBD or anemia, that have different monitoring intervals, which is potentially confusing. Some guidelines recommended testing for electrolyte disturbances, which usually develop in later CKD stages, so that it seems sensible to focus more extensive laboratory testing on patients with moderate or severe CKD. Although nephrotoxic medication can be an important risk factor for CKD progression, only BCMA and ACP recommended regular medication reviews. Blood pressure monitoring was not formally recommended by most guidelines except for HAS and BCMA, although almost all guidelines recommended specific blood pressure targets.

#### Referral criteria

Referral criteria often reflected the structure of the healthcare system and availability of resources and services. Early referral to specialist nephrology services has been linked to reduced hospitalization and mortality and increased quality of life, but was defined as more than 6 months before dialysis [33]. Because of the protracted course of CKD and low probability of most patients with CKD to progress to ESRD, only few patients with specific underlying conditions will benefit from referral to nephrologist specialty care in early CKD [34]. No longitudinal prospective studies have been conducted in the large population of patients with early CKD to assess if referral can slow CKD progression or prevent the occurrence of complications and comorbidities in this group.

Some guidelines described interdisciplinary care, but generally, no distinction was made between referral for evaluation of CKD diagnosis and ruling out kidney specific disease like glomerulonephritis, versus continuous interdisciplinary care. Main referral criteria across guidelines were refractory hypertension and progressive or advanced CKD (G4,5). Referral intervals or criteria for determining these are not proposed.

Several guidelines state that patient preferences and comorbidities should be considered when referring patients. Formal criteria for non-referral are proposed by none of the guidelines. An important unaddressed issue in all guidelines is the definition of specific referral criteria for elderly patients (80+) or nursing home residents who are unlikely to benefit from referral although CKD prevalence is high in this population. Indiscriminate application of referral criteria in this population, could lead to substantial capacity problems with respect to the nephrology workforce and may not be feasible or desirable from a public health perspective [35, 36].

#### Blood pressure

Hypertension control is important to prevent progression of CKD and all guidelines recommended blood pressure below 140/90 mmHg, with lower reference values of 130/80 for patients with diabetes or albuminuria. Although it was obvious from the context that blood pressure monitoring was expected in all guidelines, only HAS, ACP and BMCA explicitly mentioned blood pressure measurements in their monitoring recommendations.

#### Anemia

Anemia is a complication of CKD that becomes more prevalent with CKD progression. NICE recommends using a lower cut-off value of < 6,8 moll/l (11 g/dl) for diagnosing anemia, corresponding with the WHO-definition of moderate anemia, whereas KDIGO’s higher cut-off corresponds to WHO mild anemia [25, 28, 37]. Recommended monitoring frequency is somewhat lower than for GFR.

#### Other subjects

Most patients with CKD are multimorbid and the presence of CKD has implications for management of comorbid conditions. Therefore the most common associated problems should be addressed in the guideline. However, recommendations of management of comorbid conditions varied widely between the guidelines. This is a barrier for integrated management of patients with CKD.

### Strengths and limitations

Although we believe that we have not missed an important guideline on the topic and have searched in several languages, we cannot exclude language bias. We have excluded guidelines for CKD and diabetes and guidelines addressing specific issues to ensure readability and conciseness.

The AGREE-II instrument is a valuable tool to assess the methodological quality of clinical practice guidelines, but does not address content-related quality considerations such as quality of the evidence base, or applicability and acceptability of the recommendations for clinicians and patients.

Therefore, some guidelines are user-friendly for clinicians, but do not attain high scores on many of the AGREE-II items. Examples are BCMA and UMHS guidelines which provide summary tables and comprehensive overviews of management options at a glance.

### Directions for future research and guideline development

Currently, a research gap exists regarding the natural history of CKD in the general population, particularly in the elderly, and regarding the effectiveness and benefits of monitoring and treatment recommendations on preventing relatively rare but clinically important outcomes like ESRD. Research mostly addresses patients with advanced CKD or in secondary and tertiary care. Findings in these selected subgroups cannot be indiscriminately applied to the CKD population in primary care. This population, consisting mostly of elderly patients with slightly or moderately diminished kidney function, many of whom remain undiagnosed or are multimorbid with limited life expectancy and are therefore not likely to benefit from more intensive treatment or monitoring [32, 36]. These considerations are especially important regarding decisions about information, monitoring, treatment intensity and referral. CKD-stage or GFR may not always be the most appropriate criteria for decision making. A summary of recommendations for future guideline updates is provided in Table 11.

**Table 11:** Recommendations for future guidelines on CKD

1	Recommendations should specify how to consider age, multimorbidity, risk of progression, life expectancy, health goals and quality of life.
2	Recommendations on referral should distinguish between interdisciplinary or co-treatment and one-time consultations for specific problems or to rule out specific kidney diseases.
3	Guidelines should be comprehensive and include management recommendations for common CKD-related problems usually solved in primary care.
4	All relevant options including the option of abstaining from diagnosis or therapy should be incorporated in the guideline.
5	Increase involvement of stakeholders and target users, particularly non-nephrologists in the development process.
6	Implications for cost and resources in the healthcare system should be considered when formulating recommendations.
7	Facilitators and barriers to implementation and adoption of the guideline in clinical practice should be identified and analyzed and the results should be incorporated during the guideline development process.
8	A procedure and timeframe for updating the guideline should be specified.

*CKD* chronic kidney disease

## Conclusions

Clinical Practice Guidelines are increasingly issued by various stakeholders to promote quality of care. The KDIGO guideline on diagnosis and management of CKD has been adapted in many countries and served as model for most guidelines included in this review. There was substantial variation in the quality of the guideline development process.

Although there is good agreement on most core recommendations, the scope of recommendations issued by the guidelines varied significantly. Many recommendations for management of CKD rely on primarily on consensus. The care for CKD in multimorbid patients might require more individualization based on patient preferences and circumstances than can be reflected by guideline recommendations based primarily on measurement of kidney function. Since subtle differences can have a significant impact on health resource utilization and increase burden of disease in affected patients, careful implementation and evaluation of benefits and harms in every health care system is warranted.

## Additional file

Additional file 1: Compliance of different guidelines with AGREE-II. Description of how the included guidelines conform to AGREE-II items [22]. ACP: American College of Physicians, BMCA: British Columbia Medical Association, CEBAM: Belgian Centre for Evidence Based Medicine Cochrane Belgium, HAS: Haute Autorité de Santé, KDIGO: Kidney Disease Improving Global Outcomes, KHA-CARI: Kidney Health Australia – Caring for Australasians with Renal Insufficiency, NICE: National Institute of Health and Care Excellence, UMHS: University of Michigan Health System, VA-DoD: Veterans Affairs, Department of Defence. (DOCX 19 kb)

## Abbreviations

(e)GFR: (estimated) glomerular filtration rate
ACP: American College of Physicians
AEZQ: Ärztliches Zentrum für Qualität in der Medizin [*German Agency for Quality in Medicine*]
AGREE: Appraisal of Guidelines for Research and Evaluation
AWMF: Arbeitsgemeinschaft der Wissenschaftlichen Medizinischen Fachgesellschaften [*common working group of scientific medical Specialty Associations*]
BCMA: British Columbia Medical Association
CEBAM: Belgian Centre for Evidence Based Medicine
CKD: chronic kidney disease
CKD-MBD: chronic kidney disease – mineral and bone disorder
CRD: Centre for Reviews and Dissemination
ERSD: End stage renal disease
G-I-N: Guidelines International Network
HAS: Haute Autorité de Santé
KDIGO: Kidney Disease Improving Global Outcomes
KHA-CARI: Caring for Australasians with Renal Insufficiency
MeSH: Medical Subject Headings
NHS: National Health Service (United Kingdom)
NICE: National Institute of Health and Care Excellence
UMHS: University of Michigan Health System
USPSTF: United States Preventive Services Task Force
VA-DoD: United States Department of Veteran’s Affairs – United States Department of Defence
