# Supplementary material for: Diagnosis and management of non-dialysis chronic kidney disease in ambulatory care: a systematic review of clinical practice guidelines
Source: BMC Nephrol. 2018 Oct 11;19:258. doi: 10.1186/s12882-018-1048-5 (PMC6180496; doi:10.1186/s12882-018-1048-5)
Supplement: Supplementary file 1 — Compliance of different guidelines with AGREE-II. Description of how the included guidelines conform to AGREE-II items [22]. ACP: American College of Physicians, BMCA: British Columbia Medical Association, CEBAM: Belgian Centre for Evidence Based Medicine Cochrane Belgium, HAS: Haute Autorité de Santé, KDIGO: Kidney Disease Improving Global Outcomes, KHA-CARI: Kidney Health Australia – Caring for Australasians with Renal Insufficiency, NICE: National Institute of Health and Care Excellence, UMHS: University of Michigan Health System, VA-DoD: Veterans Affairs, Department of Defence. (DOCX 19 kb) [file 12882_2018_1048_MOESM1_ESM.docx]

| **AGREE-II-Items** | | **Conformity of guidelines with AGREE-II items** | | |
| --- | --- | --- | --- | --- |
|  | | **not reported (1-2 pnt)** | **incompletely reported (3-5 pnt)** | **completely reported (6-7 pnt)** |
| **Scope and purpose** | |  |  |  |
| The overall objective(s) of the guideline is (are) specifically described. | |  | NICE, ACP, BCMA, HAS | KDIGO, CEBAM, KHA-CARI, UMHS, VA-DoD |
| The clinical question(s) covered by the guideline is (are) specifically described. | | UMHS | BCMA, CEBAM, KHA-CARI, HAS | NICE, KDIGO, ACP, VA-DoD |
| The population (patients, public, etc.) to whom the guideline is meant to apply is specifically described. | |  | KHA-CARI | NICE, KDIGO, ACP, BCMA, CEBAM, UMHS, HAS, VA-DoD |
| **Stakeholder involvement** | |  |  |  |
| The guideline development group includes individuals from all the relevant professional groups. | | ACP | BCMA, CEBAM, HAS, KHA-CARI | NICE, KDIGO, UMHS, VA-DoD |
| ***Specifically:*** | | | | |
|  | *no involvement of primary care physicians* | ACP |  |  |
|  | *involvement of health care workers other than physicians* | ACP, KDIGO, CEBAM, BCMA, UMHS |  |  |
|  | *no methodologists involved* | ACP, HAS, CEBAM, BCMA, UMHS |  |  |
|  | *expertise and/or affiliations of development group members not reported* | KHA-CARI, ACP |  |  |
| The views and preferences of the target population (patients, public, etc.) have been sought. | | ACP, BCMA, VA-DoD, CEBAM, UMHS | KDIGO, KHA-CARI, HAS | NICE |
| The target users of the guideline are clearly defined. | | ACP, BCMA, KHA-CARI | NICE, UMHS | KDIGO, VA-DoD, CEBAM, HAS |
| **Rigour of development** | |  |  |  |
| Systematic methods were used to search for evidence. | | BCMA | HAS | NICE, KDIGO, ACP, KHA-CARI, VA-DoD, CEBAM, UMHS |
| The criteria for selecting the evidence are clearly described. | | BCMA, KHA-CARI, HAS | NICE, ACP, CEBAM, UMHS | KDIGO, VA-DoD |
| The strengths and limitations of the body of evidence are clearly described. | | BCMA, CEBAM, HAS | ACP, KHA-CARI, VA-DoD, UMHS | NICE, KDIGO |
| The methods for formulating the recommendations are clearly described. | | BCMA, ACP, KHA-CARI, HAS | KDIGO, VA-DoD, CEBAM, UMHS | NICE |
| The health benefits, side effects, and risks have been considered in formulating the recommendations. | | CEBAM | NICE, BCMA, KHA-CARI, VA-DoD, HAS, UMHS, KDIGO | ACP |
| There is an explicit link between the recommendations and the supporting evidence. | | HAS, UMHS | BCMA, KHA-CARI, CEBAM | NICE, ACP, VA-DoD, KDIGO |
| The guideline has been externally reviewed by experts prior to its publication. | | BCMA, ACP, KHA-CARI, VA-DoD | NICE, CEBAM, HAS, UMHS, KDIGO |  |
| A procedure for updating the guideline is provided. | | BCMA, ACP, KHA-CARI, HAS, UMHS | NICE, VA-DoD, CEBAM, KDIGO |  |
| **Clarity of presentation** | | | | |
| The recommendations are specific and unambiguous. | |  | BMCA, VA-DoD, CEBAM, UMHS, HAS | NICE, KDIGO, ACP, KHA-CARI |
| The different options for management of the condition or health issue are clearly presented. | | KHA-CARI, HAS | ACP, VA-DoD, CEBAM, UMHS | NICE, KDIGO, BMCA |
| Key recommendations are easily identifiable. | |  | NICE | KDIGO, BMCA, ACP, KHA-CARI, VA-DoD, CEBAM, UMHS, HAS |
| **Applicability** | |  |  |  |
| The guideline describes facilitators and barriers to its  application | | BMCA, ACP, KHA-CARI, VA-DoD, UMHS, HAS | NICE, KDIGO, CEBAM |  |
| ***Specifically:*** | |  |  |  |
|  | *description of tools for barrier analysis* | KDIGO, NICE, ACP, BCMA, CEBAM, HAS, KHA-CARI, UMHS, VA-DoD |  |  |
| The guideline provides advice and/or tools on how the recommendations can be put into practice. | | BMCA, ACP, VA-DoD | NICE, KDIGO, KHA-CARI, CEBAM, UMHS, HAS |  |
| The potential resource implications of applying the  recommendations have been considered. | | BMCA, ACP, KHA-CARI, VA-DoD, UMHS, HAS | KDIGO, CEBAM | NICE |
| The guideline presents monitoring and/ or auditing criteria. | | ACP, KHA-CARI, UMHS, HAS | BMCA, VA-DoD, CEBAM | NICE, KDIGO |
| **Editorial independence** | |  |  |  |
| The views of the funding body have not influenced the content  of the guideline. | | VA-DoD, HAS | KDIGO, ACP, KHA-CARI, BCMA, UMHS | NICE, CEBAM |
| Competing interests of guideline development group members  have been recorded and addressed. | | BCMA, HAS | VA-DoD | ACP, CEBAM, KDIGO, KHA-CARI, NICE, UMHS |
